# PLZF Mediates the PTEN/AKT/FOXO3a Signaling in Suppression of Prostate Tumorigenesis

**DOI:** 10.1371/journal.pone.0077922

**Published:** 2013-12-10

**Authors:** JingPing Cao, Shu Zhu, Wei Zhou, Jie Li, Chang Liu, HanQing Xuan, Jie Yan, Lin Zheng, LiXin Zhou, JianXiu Yu, GuoQiang Chen, YiRan Huang, Zhuo Yu, LiXin Feng

**Affiliations:** 1 Institute of Health Sciences, Shanghai Institutes for Biological Sciences, Chinese Academy of Sciences & Shanghai Jiao Tong University School of Medicine, Shanghai, China; 2 Laboratory for Germ Cell Research, Institute of Medical Sciences, Shanghai Jiao Tong University School of Medicine (SJTU-SM), Shanghai, China; 3 Department of Pathophysiology, Key Laboratory of Cell Differentiation and Apoptosis of Chinese Ministry of Education, SJTU-SM, Shanghai, China; 4 Department of Urology, Renji Hospital, SJTU-SM, Shanghai, China; 5 Department of Pathology, SJTU-SM, Shanghai, China; 6 Laboratory of Tumor Suppressor Genes and miRNAs, Department of Biochemistry and Molecular Cell Biology, SJTU-SM, Shanghai, China; 7 Department of Biochemistry and Molecular & Cellular Biology, Georgetown University Medical Center, Washington DC, United States of America; Winship Cancer Institute of Emory University, United States of America

## Abstract

Promyelocytic leukemia zinc finger (PLZF) protein expression is closely related to the progression of human cancers, including prostate cancer (PCa). However, the according context of a signaling pathway for PLZF to suppress prostate tumorigenesis remains greatly unknown. Here we report that PLZF is a downstream mediator of the PTEN signaling pathway in PCa. We found that PLZF expression is closely correlated with PTEN expression in a cohort of prostate cancer specimens. Interestingly, both PTEN rescue and phosphoinositide 3-kinase (PI3K) inhibitor LY294002 treatment increase the PLZF expression in prostate cancer cell lines. Further, luciferase reporter assay and chromatin immunoprecipitation assay demonstrate that FOXO3a, a transcriptional factor phosphorylated by PI3K/AKT, could directly bind to the promoter of PLZF gene. These results indicate that PTEN regulates PLZF expression by AKT/FOXO3a. Moreover, our animal experiments also demonstrate that PLZF is capable of inhibiting prostate tumorigenesis *in vivo*. Taken together, our study defines a PTEN/PLZF pathway and would shed new lights for developing therapeutic strategy of prostate cancer.

## Introduction

Prostate cancer (PCa) is the most frequently occurred cancer and the second most common cause of cancer mortality of male [1], whose progression and pathogenesis have not been well studied. Phosphatase and tensin homolog deleted on chromosome 10 (PTEN), a classic tumor suppressor, is frequently mutated or deleted in human prostate cancer and is associated with advanced prostate cancer [2], [3], [4]. Animal models also demonstrated that the loss of PTEN is strongly correlated to prostate cancer initiation and malignancy [5], [6]. PTEN is best known as an antagonist of phosphoinositide 3-kinase (PI3K)/AKT signaling pathway, a major component of the pathological avenues for a wide range of cancers development, including prostate cancer [7]. The PI3K/AKT signaling is involved in the regulation of cellular proliferation and survival [8]. Uncontrolled activation of this pathway, mainly caused by the loss-of-function of PTEN, accounts for cellular transformation and cancer development in prostate [7]. However, the detailed mechanisms at transcriptional levels remain largely unknown. We recently knocked out PTEN in spermatogonial stem cells (SSCs) and observed tumorigenesis of SSCs (see Figure S1) associated with loss of SSC marker promyelocytic leukemia zinc finger protein (PLZF) (data not shown). Besides this, recent studies also suggested that PLZF expression is regulated by PTEN activation [9], [10]. Based on these findings, we thought that PLZF might be involved in the PTEN signaling in the regulation of prostate tumorigenesis. Interestingly, we found that indeed PLZF expression is positively correlated with PTEN in human PCa samples and can be influenced by the treatment of PI3K inhibitor and AKT activation in PCa cell lines.

To understand the signaling mechanism how PLZF is regulated by the PTEN/PI3K/AKT pathway, we analyzed PLZF promoter to find its potential transcriptional regulators coupled to the PI3K/AKT pathway. We identified seven putative binding sites for forkhead transcription factor FOXO3a, which is one of the downstream targets of AKT [11]. Phosphorylation of FOXO3a by activated AKT promotes its nucleic export, thus blocking the binding of FOXO3a to the promoter of its downstream genes, which ultimately impeded its regulation of cell proliferation and survival [12]. It has been demonstrated that FOXO3a, the most highly expressed FOXO family members in prostate cancer cells, is largely deregulated with the progression of the disease [13]. Our further experiments confirmed that PLZF promoter can be regulated by FOXO3a. Thus, PLZF is a new target of the PTEN/PI3K/AKT/FOXO3a signaling. Furthermore, our animal experiments also demonstrated that PLZF is capable of inhibiting tumorigenesis of a PTEN-null PCa cell line. Taken together, these findings indicate that PLZF works at the downstream of the PTEN signaling in suppression of PCa tumorigenesis.

## Results

### PLZF expression is positively correlated with PTEN in prostate cancer

**Figure 1:**
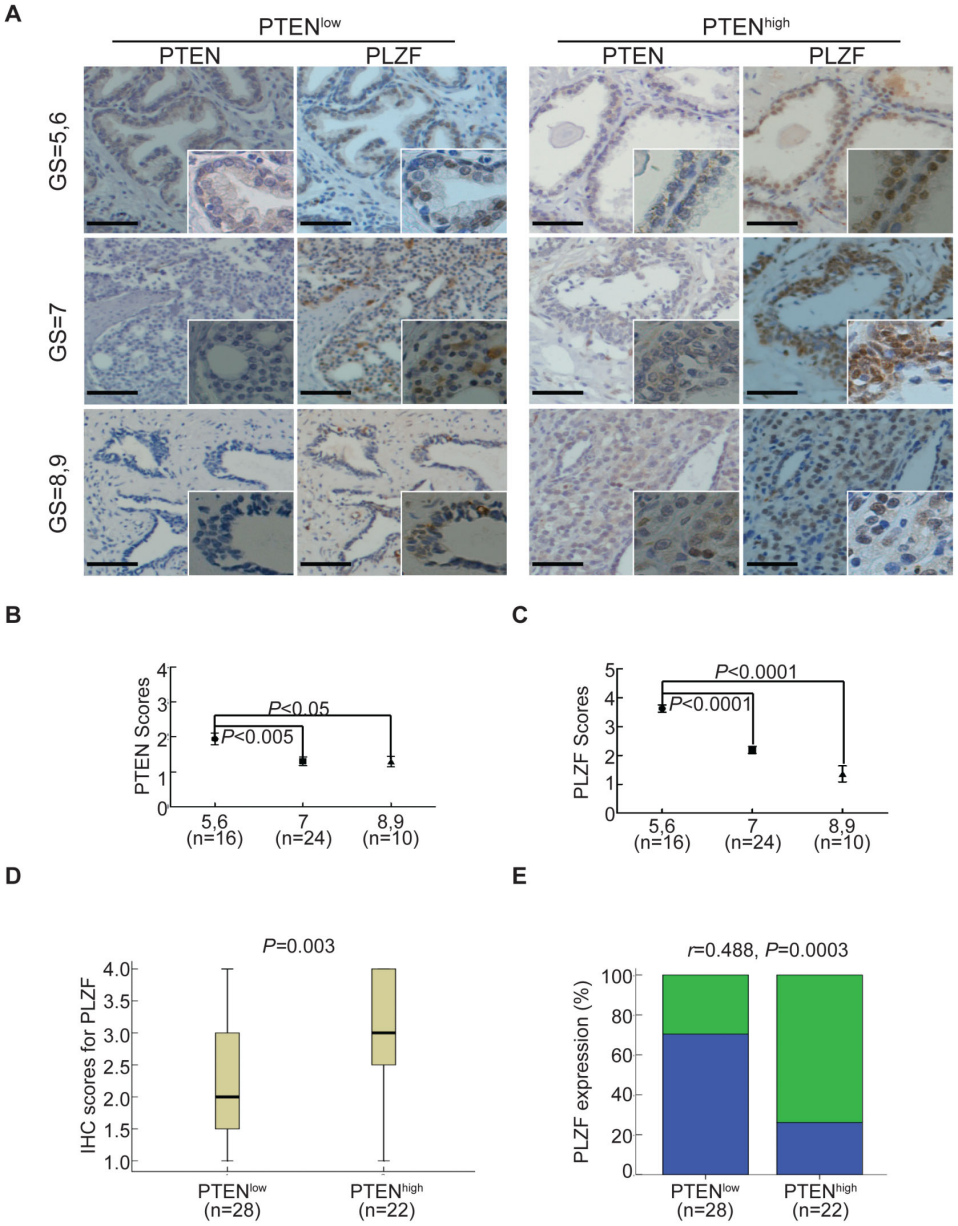
Correlation of PTEN and PLZF expressions in prostate cancer tissues. A. The representative immunohistochemical staining for PTEN and PLZF of human prostate cancer specimens of low (GS = 5, 6), moderate (GS = 7) and high grades (GS = 8, 9) by the Gleason score. Scale bar, 50 µm. B, C. The mean staining scores of PTEN (B) and PLZF (C) were evaluated in low, moderate and high grades of prostate cancer specimens. Data were shown as mean ± SD. The *P* values for comparison between line-linked groups were gotten by Student's *t*-test. D. Box plot of PLZF staining score in tumors with low and high PTEN expression. Horizontal lines represent the median; the bottom and top of the boxes represent the 25th and 75th percentiles, respectively; and the vertical bars represent the range of data. Data were analyzed by Pearson's chi-square test. E. The percentage of tumors with high and low PLZF expression in the PTEN^high^ and PTEN^low^ groups of subjects. Data were analyzed by Spearman-rank correlation.

Since our previous study has indicated that PLZF expression may be correlated with PTEN in spermatogonial stem cells, we attempted to confirm the correlation in prostate cancer. For this purpose, we first performed immunohistochemical staining to detect the expression pattern of PTEN and PLZF of 50 prostate cancer specimens, which were divided into low, moderate, and high grade of subgroups according to Gleason score (GS) ranging from 5 to 9 (Table S1), as described in Materials and methods. The representative photomicrographs from 6 cases of prostate cancer for PTEN and PLZF staining are shown in Figure 1A. In each subgroup, PLZF staining was relatively weaker when PTEN staining was low (PTEN^low^), whereas PLZF staining was more intense when PTEN staining was high (PTEN^high^) (Figure 1A). Moreover, the staining for PTEN and PLZF were gradually reduced with increased Gleason score of prostate cancer specimens (Figure 1A). We next analyzed the relationship between PTEN/PLZF expression and the development of prostate cancer. Overall, the low-grade group had significantly higher PTEN and PLZF scores than the moderate-grade and the high-grade subgroups (Figure 1B/C), suggesting that PLZF may also play a role in the clinical progress of prostate cancer like PTEN. Of great interests, Pearson's chi-square test showed that there was a remarkable positive correlation between PTEN and PLZF expressions (Figure 1D/E). A further analysis of the correlation of PTEN and PLZF was summarized in Table 1, in which most cases exhibited higher PTEN and PLZF expressions in low-grade cancer specimens, whereas most cases exhibited lower PTEN and PLZF expressions in moderate- and high-grade cancer specimens (Table 1). All these data indicate a cooperativity of PTEN and PLZF in prostate tumorigenesis.

**Table 1:** Frequency of PLZF expression in human prostate cancer specimen.

Gleason Score			PLZF	
			low	high
<7	PTEN	low	2 (12.5%)	1 (6.25%)
		high	4 (25%)	9 (56.25%)
≥7	PTEN	low	20 (58.82%)	5 (14.7%)
		high	6 (17.65%)	3 (8.83%)

### PTEN/PI3K signaling regulates PLZF expression in prostate cancer cells

**Figure 2:**
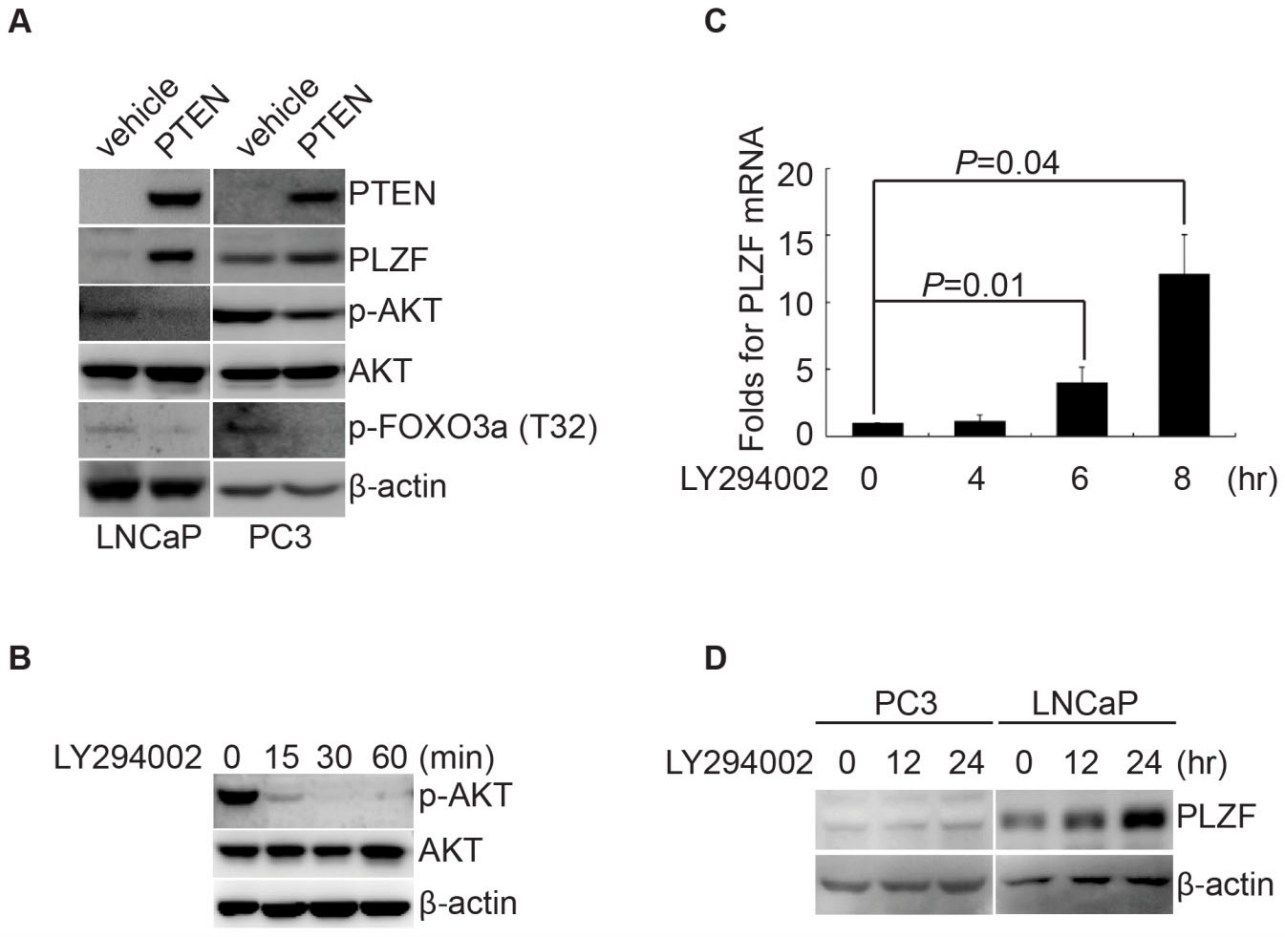
PLZF expression is influenced by PTEN in Prostate cancer cells. A. Western blotting showing that rescued expression of PTEN and the resultant induction of PLZF expression in PC3 and LNCaP cells. PC3 and LNCaP cells were transfected with PTEN or its vehicle plasmid, followed by Western blots for the proteins as indicated. B. PC3 cells were treated with LY294002 for the indicated times, and AKT and its phosphorylated form (p-AKT) were detected by western blots with β-actin as loading control. C. PC3 cells were treated with LY294002 for the indicated time. Then, PLZF mRNA was detected by Q-PCR. Folds of PLZF mRNA against cells in LY294002-treated for 0 hour were calculated, and results were shown as means with bar as SD in three independent experiments with triplicate each. The *P* values for comparison between line-linked groups were gotten by Student's *t*-test. D. PC3 cells and LNCaP cells were treated with LY294002 for the indicated time. PLZF proteins were detected by Western blots with β-actin as loading control. All experiments were repeated individually for three times.

The positive correlation between PTEN and PLZF expressions in prostate cancer tissues promoted us to speculate that PTEN regulates PLZF expression. We firstly transfected PTEN into PC3 cells and LNCaP cells, which are absent of endogenous PTEN protein expression. As expected, PTEN overexpression inhibited AKT activation and it increased PLZF expression accordingly (Figure 2A). Since PTEN is known as a lipid phosphatase that antagonizes the PI3K/AKT signaling pathway [7], we next treated prostate cancer cell lines PC3 and/or LNCaP cells with PI3K inhibitor LY294002 for hours as indicated. The results showed that LY294002 treatment significantly inhibited phosphorylation of AKT (Figure 2B), suggesting its effectiveness to inhibit PI3K/AKT activity. More interestingly, quantitative real-time PCR analysis showed that LY294002 treatment increased PLZF expression in a time-dependent manner in PC3 cells (Figure 2C). In line with this, LY294002 also increased PLZF protein in PC3 and LNCaP cells (Figure 2D). All these data propose that PTEN upregulates PLZF expression through inhibiting PI3K/AKT signaling.

### FOXO3a regulates PLZF expression in prostate cancer cells

**Figure 3:**
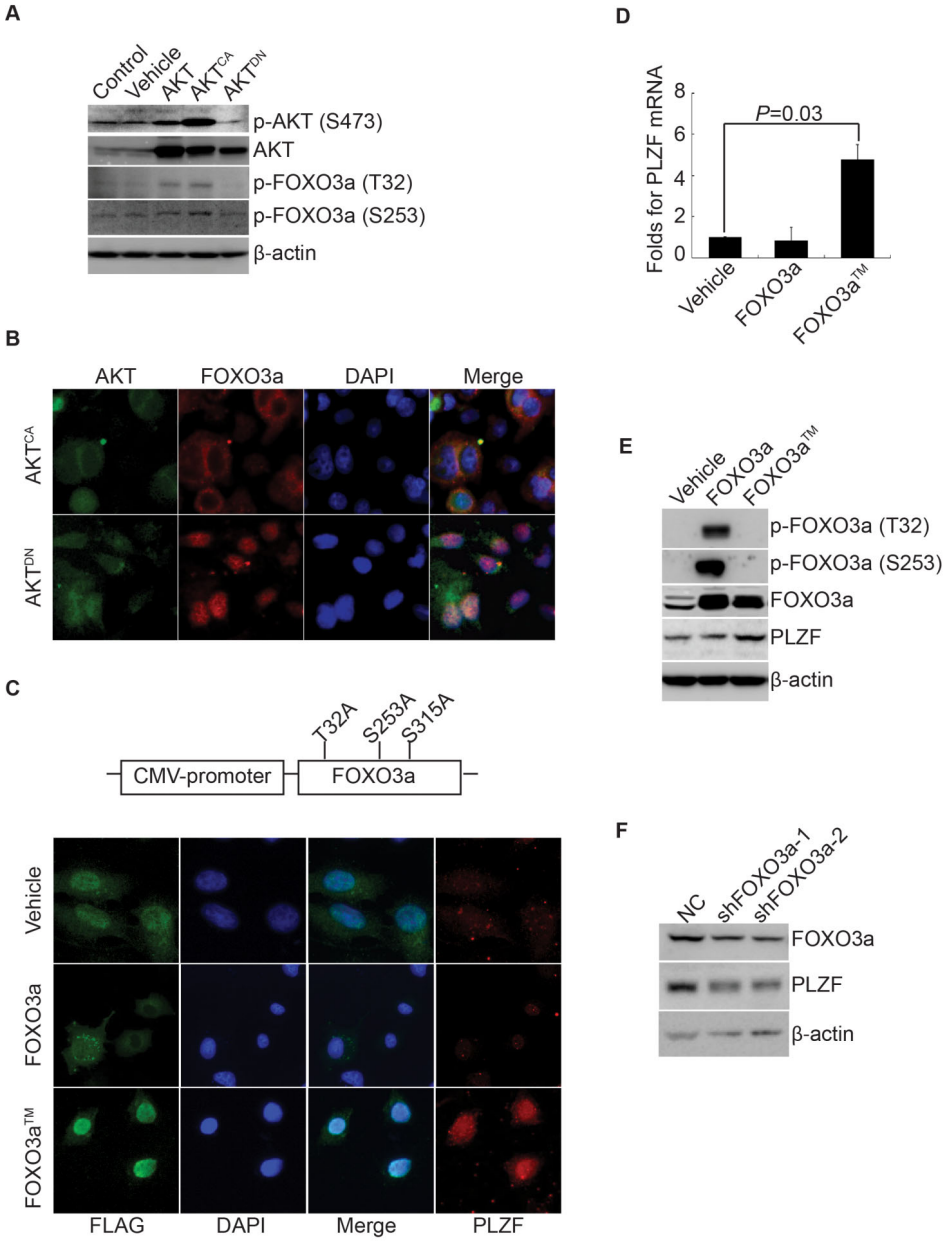
FOXO3a regulates PLZF expression in prostate cancer cells. A. Wild-type AKT, AKT^CA^, and AKT^DN^ were transfected into PC3 cells, and Western blots were performed with the proteins as indicated. B. AKT^CA^ and AKT^DN^ were transfected into PC3 cells, followed by immunofluorescent staining of AKT and FOXO3a with re-stain of DAPI. The representative images were recorded under a 40× objective. C. Flag-tagged FOXO3a and FOXO3a™ were transfected into PC3 cells, followed by immunofluorescent staining of Flag and PLZF with re-stain of DAPI. The representative images were recorded under a 40× objective. D, E. Wild-type FOXO3a and triple mutant, FOXO3a™ were transfected into PC3 cells respectively, and Q-PCR (D) and Western blots (E) were performed as indicated. In panel D, folds of PLZF mRNA against cells transfected with vehicle were calculated, and results were shown as means with bar as SD in three independent experiments with triplicate each. The *P* values for comparison between line-linked groups were gotten by Student's *t*-test. F. ShRNAs specifically against FOXO3a along with its non-specific shRNA (NC) were respectively transfected into LNCaP cells, and Western blots were performed with FOXO3a and PLZF with β-actin as loading control. All the experiments were repeated individually for three times.

Next we addressed how PTEN/PI3K/AKT regulates PLZF expression. It has been exhibited that FOXO3a, which is the most highly expressed FOXO family member in prostate cancer cells, is closely related with the progression of prostate cancer [13]. AKT was also reported to phosphorylate FOXO3a at three conserved sites (Thr32/S253/S315), thus sequestering FOXO3a in the cytoplasm from its transcriptional involvement in the nucleus [14]. Indeed, here we showed that ectopic expression of constitutively-activated AKT (AKT^CA^) increased, while dominant-negative form of AKT (AKT^DN^) decreased the phosphorylations of FOXO3a (Figure 3A). Also, AKT^CA^ expressions sequestered FOXO3a in the cytoplasm, while AKT^DN^ expression kept it in the nuclei of PC3 cells (Figure 3B). Accordingly, PTEN overexpression inhibited the phosphorylations of FOXO3a (Figure 2A). Thus, we transfected wild-type FOXO3a or its mutant (FOXO3a™) at three conserved AKT-phosphorylated sites into PC3 cells for 24 hours. Immunofluroscent staining exhibited that wild-type FOXO3a protein was scattered in the cytoplasm and FOXO3a™ was accumulated in the nucleus (Figure 3C). More intriguingly, FOXO3a™ expression significantly elevated PLZF expression (Figure 3C). Further, quantitative real-time PCR and western blot analysis demonstrated that FOXO3a™ sufficiently induced the expression of PLZF (Figure 3D/E). On the other hand, the knockdown of FOXO3a by its specific shRNA (shFOXO3a-1 and shFOXO3a-2) significantly reduced the expression of PLZF in LNCaP cells (Figure 3F). All these results strongly suggest that FOXO3a may be a mediator of the PTEN/PI3K/AKT signaling to regulate the expression of PLZF.

### FOXO3a directly binds to the promoter of PLZF gene

**Figure 4:**
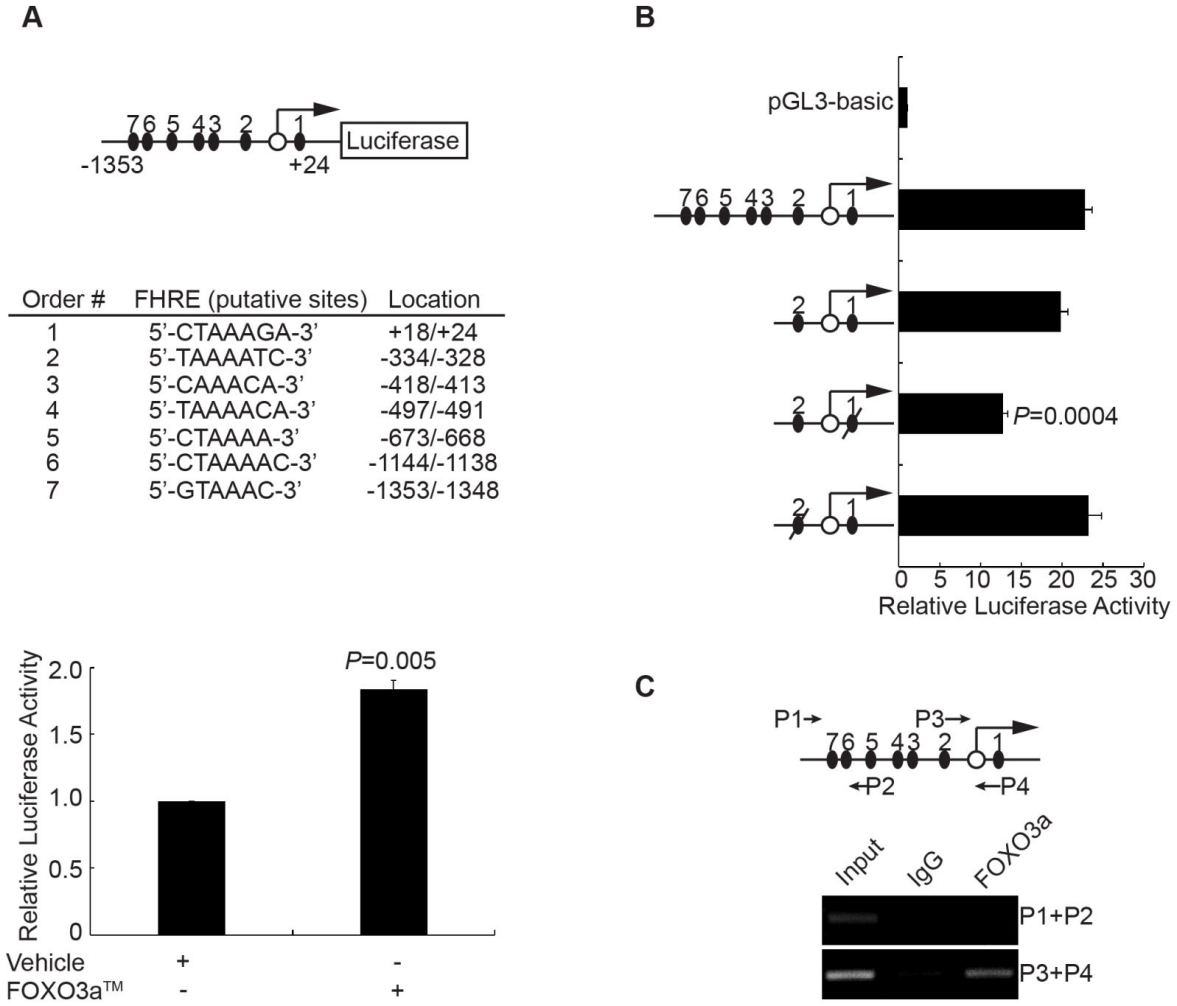
PLZF is a direct target gene of FOXO3a. A. The luciferase reporter plasmid pGL3-PLZF, shown in the top and middle panel, is driven by seven putative FHREs in PLZF promoter. In the top diagram, black ovals and the empty circle represent the putative FHREs and the transcriptional start site, respectively. PC3 cells were transfected with 100 ng of pGL3-PLZF and/or 200 ng of FOXO3a™ together with pSV40-renilla for 24 hours. The folds of the relative pSV40-Renilla-normalized FHREs-Luc activity against cells without FOXO3a™ were calculated, shown as the means with bar as SD from three independent experiments. The *P* values for comparison were gotten by Student's *t*-test. B. PC3 cells were transfected with pGL3-PLZF or its truncations or empty vector pGL3-basic as indicated in the presence of FOXO3a™ expressing vector for 24 hours. The folds of the relative pSV40-Renilla-normalized FHREs-Luc activity against cells with empty vector pGL3-basic were calculated, shown as the means with bar as SD from three independent experiments. The *P* values for comparison were gotten by Student's *t*-test. C. PC3 cells were cross-linked and lysed, followed by immunoprecipitating with anti-FOXO3a antibody or non-specific IgG. Precipitated DNAs and total lysis were amplified by PCR with primers for district between FHRE1 and FHRE2 or district between FHRE6 and FHRE7, as shown in the top panel. The ChIP assay was repeated individually for three times.

To further determine if FOXO3a regulates PLZF expression by its transcriptional function, we conducted luciferase reporter assay. Bioinformatic analysis found seven potential forkhead response elements (FHRE) within a 2.3 kb region containing the 5′ upstream sequences and the first exon of PLZF gene (Figure 4A). We subcloned the DNA fragment into the luciferase reporter vector pGL3-basic, which was co-transfected with empty vector or pcDNA3.1(+)-FOXO3a™ into PC3 cells. The results showed that FOXO3a™ dramatically increased the luciferase activity (Figure 4A). Furthermore, we constructed luciferase reporters driven by different FHREs (Figure 4B), which were co-transfected together with FOXO3a™ into PC3 cells. As shown in Figure 4B, FHRE1 and FHRE2 were sufficient for FOXO3a™-induced luciferase expression, which could be abrogated by mutations on FHRE1, indicating that FHRE1 is indispensable for the transcription of PLZF driven by FOXO3a. The chromatin immunoprecipitiation (ChIP) results also supported that FOXO3a directly bound to the promoter of PLZF in the region of FHRE1 and FHRE2 (Figure 4C). Given all these data, we conclude that FOXO3a upregulates PLZF expression by its transcriptional activity.

### FOXO3a expression is positively correlated with PLZF in prostate cancer

**Figure 5:**
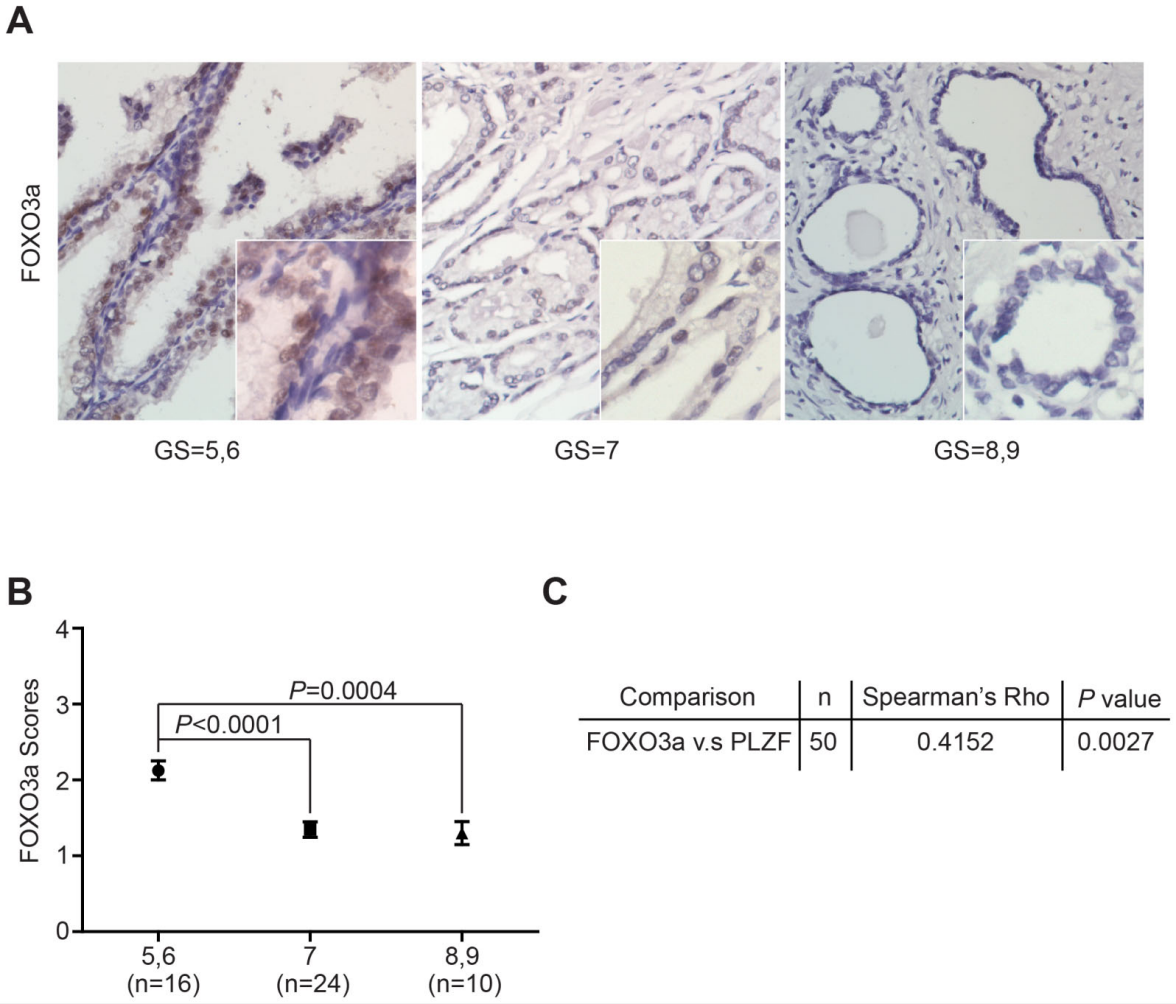
FOXO3a expressions in prostate cancer tissues. A. The representative immunohistochemical staining for FOXO3a of human prostate cancer specimens of low (GS = 5, 6), moderate (GS = 7) and high grades (GS = 8, 9) by the Gleason score. Scale bar, 50 µm. B. The mean staining scores of FOXO3a was evaluated in low, moderate and high grades of prostate cancer specimens. Data were shown as mean ± SD. The *P* values for comparison between line-linked groups were gotten by Student's *t*-test. C. The Spearman correlation of FOXO3a and PLZF expression to each other was illustrated.

Given that we have demonstrated that PTEN upregulated PLZF through FOXO3a by its transcriptional activity, it needs to be further confirmed whether this correlation also exists in the clinical specimens. As above, we detect the expression pattern of FOXO3a in the prostate cancer specimens by immunohistochemistry staining (Table S1). The representative photomicrographs from 3 cases of prostate cancer for FOXO3a staining are shown in Figure 5A. The staining for FOXO3a was reduced with increased Gleason score of prostate cancer specimens (Figure 5A). Next we analyzed the correlation between FOXO3a expression and the development of prostate cancer. Overall, the low-grade group had significantly higher FOXO3a scores than the moderate-grade and the high-grade subgroups (Figure 5B), suggesting that FOXO3a may gradually lose its function along with the progression of prostate cancer. As expected, we presented a significant positive correlation between FOXO3a and PLZF expressions by Spearman's Correlation analysis (Figure 5C). All these data present a close correlation of FOXO3a with PLZF, and indicate a cooperativity of FOXO3a and PLZF in prostate carcinogenesis.

### PLZF inhibits the tumor growth of PC3 cells in xenograft nude mice

**Figure 6:**
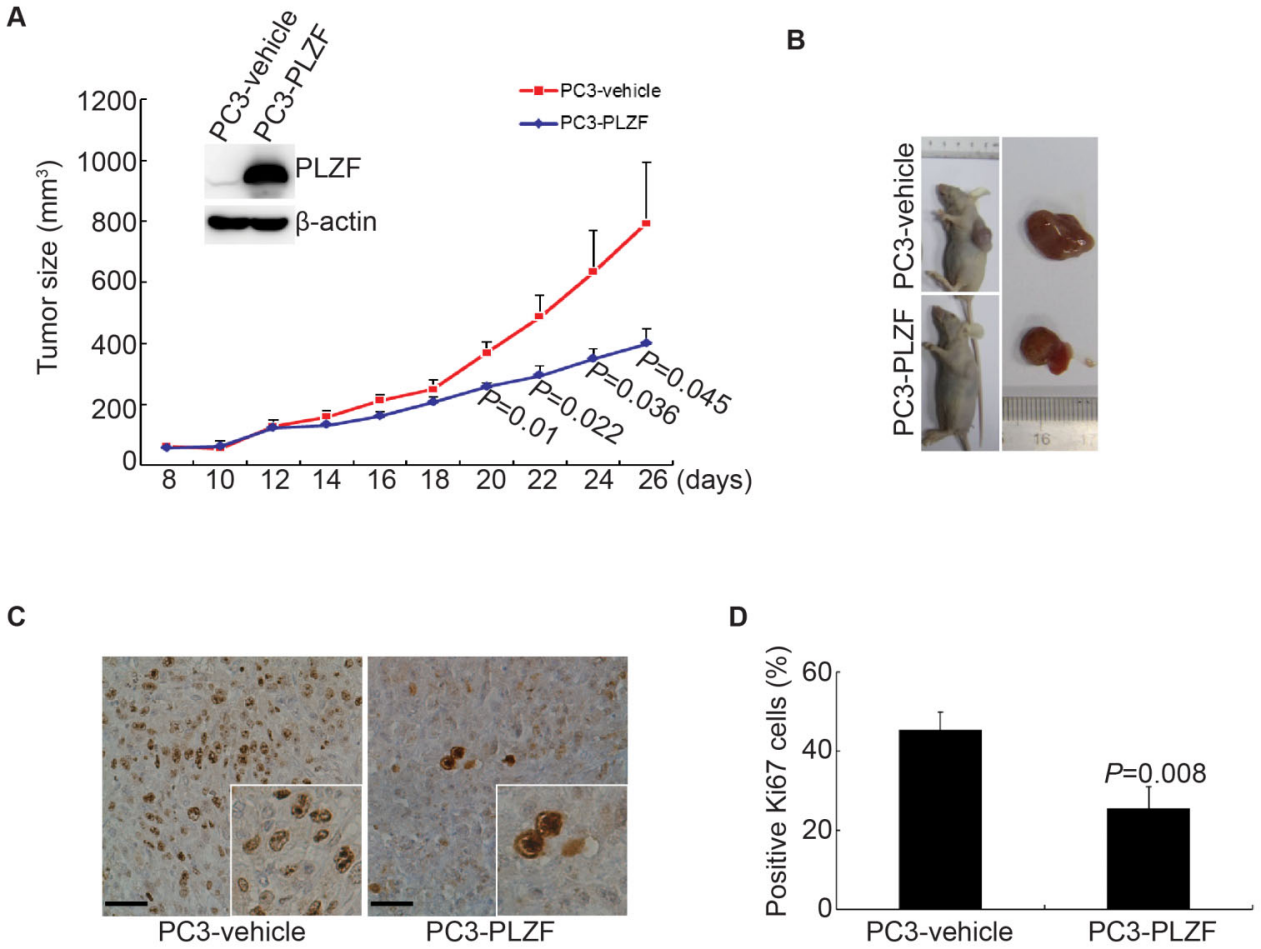
PLZF inhibits the tumor growth in nude mice. A. The tumor volumes (six mice for each group) were measured at the indicated times, shown as the means with bars as SD. The *P* values for comparison were gotten by Student's *t*-test. The inserted western blots showed PLZF expression. B. Representative macroscopic images for tumors 26 days after subcutaneous injection. C. Representative immunohistochemistry staining of Ki67 (scale bar, 50 µm) for PLZF-expressing and empty vector-carrying tumors. The representative images were recorded under a 40× objective. D. The statistical analysis of the accounts of the Ki67 positive cells was displayed on the right panel, shown as the means with bar as SD. The *P* values for comparison were gotten by Student's *t*-test.

To further investigate the potential role of PLZF *in vivo*, PC3 cells stably transfected with PLZF-expressing vector or the empty vector were subcutaneously injected into the 6-week-old male athymic nude mice. All of the mice developed the tumor after one week and the tumor volumes were measured every other day. Transplanted tumors originated from PLZF overexpression cells grew more slowly than tumors from empty-vector cells (Figure 6A). Twenty-six days later, mice were sacrificed, and tumors were picked. Figure 6B showed the representative macroscopic images for the PLZF-expressing and empty vector-carrying PC3 cells-transplanted tumors. Immunohistochemical staining for Ki67, a widely used proliferation marker, showed that PLZF-expressing tumors exhibited fewer Ki67-positive cells than the empty vector-carrying tumors (Figure 6C/D). Based on these results, we conclude that PLZF plays a critical role in suppression the tumorigenesis of PCa.

## Discussion

Many studies have demonstrated that PTEN plays an important role in the development of prostate cancer, whose genomic deletion is closely associated with poor outcome of localized and androgen-independent prostate cancer [15], [16], [17]. By FISH assay, it has been shown that homozygous deletion of PTEN is more frequently in high-grade (GS = 8,9) prostate cancer specimens than in median- (GS = 7) and low-grade (GS = 5,6) ones [18]. Besides this, several groups have provided evidences that PTEN loss along with other pathways, such as RAS/MAPK and JNK, contributes to the progression of prostate cancer, especially the metastatic process [5], [6]. However, the downstream executor of PTEN remains largely unknown. Based on our preliminary study in PTEN conditional knock-out mice and previous studies [9], [10], we hypothesized that PLZF might be the downstream modulator cooperating with PTEN in regulating the progression of prostate cancer.

To test this hypothesis, we first performed immunohistochemical staining of PTEN and PLZF in a cohort of human PCa samples. The results presented lower expressions of PTEN and PLZF in high-grade prostate cancer specimens (GS = 8,9) than in mediate-grade (GS = 7) and well-differentiated (GS = 5,6) specimens. Further statistical analysis revealed a positive correlation between PTEN and PLZF, indicating that PLZF expression is influenced by PTEN in the progression of prostate cancer. Next we confirm this correlationship of PTEN and PLZF in PCa cell lines. Restoration of PTEN in PTEN-null prostate cancer cell lines dramatically upregulated PLZF expression along with the inhibition of AKT phosphorylation, indicating that PTEN is an upstream regulator on PLZF expression. After the treatment with PI3K inhibitor, LY294002, PLZF expression is upregulated in both mRNA and protein levels in LNCaP and PC3 cells. Thus, PLZF is involved in the PTEN/PI3K/AKT pathway in PCa cells.

Importantly, we also found for the first time that PLZF is a direct target gene of FOXO3a. Within the PLZF promoter containing the 2.3 kb upstream the transcription start site and the first exon [25], we identified seven putative binding sites of the consensus forkhead response element (FHRE) A/G TAAA T/C A [26], including FHRE1 (+18∼+24), FHRE2 (−334∼−328), FHRE3 (−418∼−413), FHRE4 (−497∼−491), FHRE5 (−673∼−668), FHRE6 (−1144∼−1138), and FHRE7 (−1353∼−1348). FOXO3a™ overexpression significantly enhanced luciferase expression driven by the promoter of PLZF gene. Deletion-based luciferase reporter assay revealed that FOXO3a binding sites are located in the region of FHRE1 and FHRE2 within PLZF promoter. Individual abrogation of binding site assay demonstrated that FHRE1 is essential for FOXO3a binding on the promoter of PLZF while FHRE2 mutation does not influence significantly the luciferase expression. Chromatin immunoprecipitation assay confirmed the binding of FOXO3a is particularly located in the region of FHRE1 and FHRE2 instead of FHRE6 and FHRE7. In addition, ectopically expressed FOXO3a™ protein significantly increased PLZF expression in both mRNA and protein level in PC3 cells, and silence of FOXO3a by specific shRNA reduced PLZF expression. Furthermore, a significant positive correlation is seen in a cohort of human PCa samples between FOXO3a and PLZF expressions. Thus, we confirmed that PLZF mediates the PTEN signaling through the AKT/FOXO3a cascade. Its function in tumor suppression was further verified by xenograft animal experiments using PC3-PLZF cell line.

The promyelocytic leukemia zinc finger protein (PLZF), firstly discovered as part of the fusion protein with retinoic acid receptor-α (RARα) in acute promyelocytic leukemia (APL) [27], belongs to the family of Kruppel-type zinc finger proteins and contains a conserved POZ protein-binding domain at its N-terminal. PLZF^−/−^ mice are prone to develop defect patterning in limb and axial skeleton [28]. Besides this, PLZF^−/−^ mice displayed the disruption of osteoblastic differentiation during the early stages of pluoripotent differentiation of human mesenchymal stem cells [29]. Recently, large studies support that PLZF plays a key role in maintaining the pool of spermatogonial stem cells [10], [30]. All these data indicated that PLZF might have a significant influence on maintaining the dynamic balance of self-renewal and differentiation. Since cancer stem cells share some characteristics with embyonic stem cells/progenitors, it should be a potential field to explore the activity of PLZF in cancer stem cells.

In addition, other studies have provided evidence showing PLZF is involved in the regulation of cell cycle progression and growth suppression [21] by repressing the expression of cyclin A2, c-myc, and c-kit [22], [23], [24]. Deregulation of PLZF activity is demonstrated to be linked with the progression of multiple solid tumors including prostate cancer [19], [20], [31]. It has been shown that PLZF can directly bind to the promoter region of miR-221/222. Loss of PLZF in advanced melanoma cells unblocked miR-221/222, which in turn enhanced cell proliferation and invasion [31]. Besides microRNA, it has been shown that the transcriptional repression by PLZF is dependent on histone acetyltransferase (HAT), p300, which specifically acetlated the lysine residues in its C-terminus of zinc finger motif [32]. PLZF acetylation appears to promote its binding to DNA, thus enhancing its transcriptional repressor activity in this context. Based on these data, we believe that the loss of function of PLZF is an intriguing event during the development of solid tumors. In future studies, we should pay more attentions on the interaction network between PLZF and other DNA/protein/micorRNA, which would help us understand the role of PLZF in tumor progression more thoroughly, and shed new light on providing more clinical strategies.

**Figure 7:**
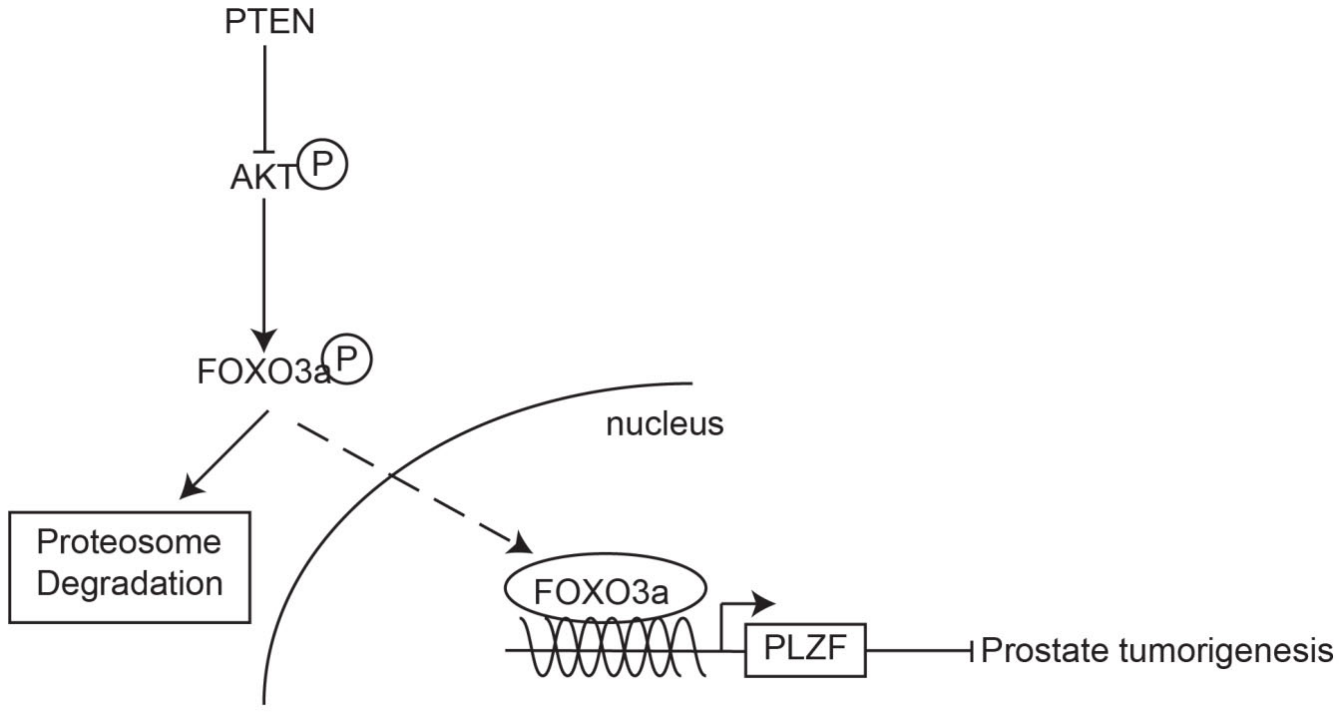
The putative PTEN/FOXO3a/PLZF signaling pathway in the development of prostate cancer.

Based on our *in vitro* and *in vivo* results, we propose that PLZF functions as a down-stream mediator of PTEN signaling and we define a signaling axis of PTEN/AKT/FOXO3a/PLZF in PCa, depicted in Figure 7. Our study underscores the importance of PLZF in suppressing prostate tumorigenesis and indicates further investigations on prostate-specific PLZF knock-out mice. Our findings may contribute to expand our knowledge on the prostate cancer progression and establish therapeutic strategies in the management of prostate cancer.

## Materials and Methods

### Ethics statement

Written informed consent was obtained from each patient for his enrollment. The study was approved by the Institutional Review Board of Renji Hospital, Jiao-Tong University School of Medicine (Shanghai, China). All clinical investigation was conducted in accordance with the principles expressed in the Declaration of Helsinki.

### Cell culture and reagents

PC3 and LNCaP cells (obtained from ATCC) were cultured in DMEM medium (GIBCO, Life Technology, NY, USA) and RPMI 1640 (GIBCO, Life Technology, NY, USA), respectively, supplemented with 10% new-born calf serum (NBCS, GIBCO), 1% penicillin/streptomycin (GIBCO), and 1% non-essential amino acid (GIBCO) in a humidified incubator in an atmosphere of 5% CO_2_, 37°C. HEK293T cells were applied to produce high titer retrovirus and cultured in DMEM medium. The PI3K inhibitor LY294002 was purchased from Cell Signaling Technology (Hitchin, United Kingdom), dissolved in DMSO according to the instruction.

### Plasmid constructs and transfection

The plasmid expressing human FOXO3a was kindly given by Chuxia Deng laboratory (NIDDK, NIH). Triple mutant of FOXO3a at three AKT phosphorylation sites (T32A/S253A/S315A) was generated by using QuikChange Lightning Site-Directed Mutagenesis Kit (Stratagene, La Jolla, CA). The mutant was then sub-cloned into pcDNA3.1(+). PLZF cDNA (NG_012140) was constructed into AAV-IRES-hrGFP. These plasmids were transfected into PC3 by Lipofectamine 2000 transfection reagent (Invitrogen, Carlsbad, CA92008, USA) according to the manufacturer's instructions. Constitutively-activated AKT (E17K) and dominant- negative AKT (R25C, S473A) were generated by site mutagenesis. Wild-type, constitutively-activated and dominant-negative forms of AKT (NG_012188) were constructed into retroviral vector pMSCV-IRES-GFP (pMIG). PTEN cDNA (NG_ 007466) was subcloned to pMIG. The indicated sequences of the promoter of PLZF were amplified from human genomic DNA (Clonetech, Otsu, Shiga, Japan) and sub-cloned into the luciferase reporter plasmid, pGL3-basic (Promega, Madison, Wisconsin, USA).

### Virus infection

Retroviruses were prepared by transient co-transfection of these plasmids with helper plasmids into 293T cells. PC3 and LNCaP cells were infected at approximately 70% confluence in DMEM supplemented with 8 µg/ml of polybrene. Forty-eight hours later, the medium was changed to DMEM medium with 10% FBS and added 4 µg/ml puromycin for 3 days to screen stable cell lines for further assay. Viruses-expressing AKT or PTEN infected into PC3 cells were generated from HEK293T by co-transfected with gag-pol/vsvg system.

### shRNA design and infection

ShRNA oligoes specifically against FOXO3a were synthesized, annealed and ligated to pSIREN-RetroQ according to the manufacturer's instruction (Clonetech). The target sequences for FOXO3a were GCTGTCACTGCATAGTCGA (shFOXO3a-1) and GCGTTCACGCACCAATTCT (shFOXO3a- 2).

### Quantitative real-time reverse Transcription-polymerase chain reaction

Total RNAs were isolated from cultured cells using TRIzol (Invitrogen, Carlsbad, CA 92008, USA). Complementary DNAs were synthesized by using the cDNA synthesis kit according to manufacturer's instructions (TaKaRa, Da-lian, China). PLZF mRNA level was quantified by real-time PCR with the double-stranded DNA dye SYBR Green PCR Master Mixture Reagents (Applied Biosystems, Warrington, UK) with the ABI PRISM 7300 system (Applied Biosystems, Foster City, CA). The specific primers were: PLZF, 5′-tggcactgacatggccgtcttc-3′ (forward) and 5′-cgtaggggtggtcgcctgtatg-3′ (reverse); β-actin, 5′-catcctcaccctgaagtaccc-3′ (forward) and 5′-agcctggatagcaacgtacatg-3′ (reverse), as an internal control.

### Luciferase assay

For the luciferase assay, FOXO3a™, along with the luciferase reporter plasmid driven by PLZF promoter fragments and pSV40-Renilla, as an internal control, were co-transfected into the PC3 cells seeded in a 12-well plate. Twenty-four hours later, cells were lysed and analyzed for the luciferase assay according to the manufacture's instruction (Promega).

### Chromatin immunoprecipitation (ChIP)

PC3 cells were cross-linked with 1% formaldehyde at 37°C for 10 minutes, and cells were scraped down in 500 µl RIPA lysis buffer (Beyond, Shanghai, China) supplemented with PMSF (Beyond) and protease inhibitor cocktail (Sigma-Aldrich Corp, St. Louis, MO, USA). Then DNA of the cells was sonicated and sheared to small fragments of 500–1000 bp with Sonicator ultrasonic processor (Misonix, Farmingdale, NY). Subsequently, the supernatant of the sonicated cells was collected, diluted and precleared by protein A+G agarose (Calbiochem). Furthermore, anti-human FOXO3a antibody (Cell Signaling Technology, Hitchin) was added to the supernatant for immunoprecipitation with normal pre-immuned mouse IgG (Santa Cruz Biotechnology) as a normal control. After overnight incubation, the protein A+G agarose were added and incubated for additional 3 hours and then washed with low-salt, high-salt and LiCl buffers serially, then the immunoprecipitated DNA was retrieved by 5 M NaCl at 65°C for 4 hours and purified with a DNA purification kit (Qiagen). PCR for the FHRE in the promoter was performed with specific primers: 5′-gcagactcctgctgggctga-3′ (P1, forward) and 5′-gctgaaggccaggcggtaga-3′ (P2, reverse); 5′-tccccatgcactcctgtcctct-3′ (P3, forward) and 5′-tccgggcttcgcctgacatcta-3′ (P4, reverse).

### Western Blot

Protein extracts were collected by ice-cold lysis buffer containing 50 mM Tris-HCl (pH 7.4), 1 mM EDTA, 150 mM NaCl, 1% sodium deoxycholate, 0.1% SDS, 10 mM sodium fluoride, 1 mM sodium orthavanadate, and 1% protease inhibitor cocktail (Sigma-Aldrich Corp). Extracted proteins (20 µg) were separated by SDS-PAGE gel, and transferred onto PVDF membrane (Millipore, MA, USA). The membranes were probed with the antibody specific for PLZF (1∶200, R&D systems) and antibodies purchased from Cell Signaling Technology (Hitchin) specific for PTEN, p-AKT (Ser473), AKT, p-FOXO3a(Thr32), p-FOXO3a(Ser253), p-FOXO3a (Thr318/321), FOXO3a, and β-actin, which were diluted in 1∶1000. The signals were detected by the appropriate secondary antibodies and visualized by ECL detection system (Millipore). The images were obtained by LAS-4000 mini system (FUJIFILM, Minato-ku, Tokyo, Japan).

### Immunofluorescence

For immunofluorescence, the transfected PC3 cells were fixed in 4% paraformaldehyde for 10 min and incubated with antibodies against phosho-FOXO3a (diluted 1∶100, Cell Signaling Technology, Hitchin). After washing with 1×PBS buffer, cells were incubated with Cy3- conjugated secondary antibodies together with DAPI to stain nuclei. The slides were mounted and viewed under a fluorescence microscope (Nikon ECLIPSE Ti-S).

### Immunohistochemistry

All 50 prostate cancer specimens collected from April 2003 to January 2010 by the Department of Urology at Renji Hospital, Jiao-Tong University School of Medicine (Shanghai, China), histologically graded according to the Gleason method [33], were separated into low-, moderate-, and high-grade subgroups, defined as cases with a combined GS = 5,6, GS = 7 or GS = 8,9, respectively. Serial sections of these specimens from formalin-fixed and paraffin-embedded human Prostate cancer tissues were deparaffinised in xylene and rehydrated in graded ethanol solutions. Sections were pretreated in heated sodium citrate buffer (0.01 M, pH 6.0) for 15 minutes in 100°C followed by blocking the endogenous peroxidase with 0.3% hydrogen peroxide, and being incubated with PBS (0.1 M, pH 7.0) containing 10% donkey serum (Jackson ImmunoResearch Europe Ltd, Newmarket, Suffold, UK). Following overnight incubation with the antibodies including PLZF (diluted at 1∶100, R&D), PTEN (diluted at 1∶200, Cell Signaling Technology), or FOXO3a antibody (diluted at 1∶100, Cell Signaling Technology) at 4°C, the sections were stained with the corresponding HRP-conjugated secondary antibody (KIT-9719 and KIT-9707, Mai-xin Biotechnology, Fuzhou, China). The visualization was performed with a diaminobenzidine (DAB) reaction (Mai-xin), resulting in a brown staining of structures containing the epitope. Slides were counterstained with hematoxylin before permanent mounting and then evaluated under a light microscope (Carl Zeiss, Maple Grove, MN, USA). IHC staining scores of PLZF and PTEN were judged depending on the staining intensity, which was scored by using a 4-tier system as negative (score = 1), weak (score = 2), moderate (score = 3) and strong (score = 4) staining.

### Animal experiments

PLZF-overexpressing and its vector-control PC3 cells (2×10^6^), harvested, washed and resuspended in 100 µl serum-free DMEM, were injected subcutaneously into the left flank of the six-week old athymic nude-mice (six mice for each group) obtained from SLAC Inc. (Shanghai, China). When the tumors were detectable one week after injection, they were measured every two days with venire calipers for three more weeks. The tumor volume were calculated with the following formula: 1/2(the shortest diameter)^2^×(the longest diameter). All mice were sacrificed and all visible tumors were excised and separated for immunohistochemistry staining. All of the animals care and experimental protocols was approved by the animal committee of Shanghai Jiao-Tong University School of medicine (Shanghai, China).

### Statistical analysis

Pearson's chi-square test and Spearman-rank correlation were employed to evaluate the results of immunohistochemistry. All statistical analyses were conducted with SAS Enterprise software (SAS Institute, Cary, NC). The values were exhibited as mean ± SD. The Student's *t*-test was used for statistical analysis between two groups. Significant level was set at *P*<0.05.

## Supporting Information

Figure S1 **The formation of tumor in** ***PTEN*** **knock-out mice.** The representative images of the tumor formed in one flank of testis (arrow) in *PTEN* knock-out mice.
(TIF)

Table S1 **Summary of PTEN, PLZF and FOXO3a staining scores in human PCa samples.**
(PDF)
