# Supplementary figures and images for: PLZF Mediates the PTEN/AKT/FOXO3a Signaling in Suppression of Prostate Tumorigenesis
Source: PLoS One. 2013 Dec 10;8(12):e77922. doi: 10.1371/journal.pone.0077922 (PMC3858220; doi:10.1371/journal.pone.0077922)

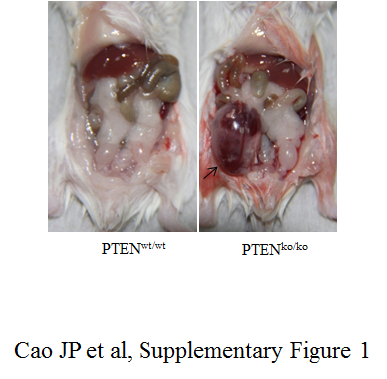

Supplement: Figure S1 — The formation of tumor in PTEN knock-out mice. The representative images of the tumor formed in one flank of testis (arrow) in PTEN knock-out mice. (TIF) [file pone.0077922.s001.tif]
