# Supplementary material for: PLZF Mediates the PTEN/AKT/FOXO3a Signaling in Suppression of Prostate Tumorigenesis
Source: PLoS One. 2013 Dec 10;8(12):e77922. doi: 10.1371/journal.pone.0077922 (PMC3858220; doi:10.1371/journal.pone.0077922)
Supplement: Table S1 — Summary of PTEN, PLZF and FOXO3a staining scores in human PCa samples. (PDF) [file pone.0077922.s002.pdf]

Table S1. Summary of PTEN, PLZF and FOXO3a staining scores in human PCa samples.

Case No.	Gleason score	PTEN score	PLZF score	FOXO3a score
07-12140	2+3	2	3	3
08-10790	2+3	3	4	3
04-151	2+3	2	4	2
07-14807	3+2	3	3	2
07-2194	3+2	2	4	2
05-12053	3+3	1	3	2
07-4581	3+3	2	4	2
07-622	3+3	1	3	2
07-6828	3+3	2	4	2
07-8579	3+3	2	4	2
07-8639	3+3	2	3	2
08-00404	3+3	3	4	1
08-05447	3+3	2	4	2
08-17156	3+3	1	4	3
09-10542	3+3	2	3	3
10-01076	3+3	2	4	2
05-12365	3+4	2	3	2
05-9602	3+4	1	3	1
04-2707	3+4	1	3	2
07-1390	3+4	1	3	1
07-1640	3+4	2	3	2
07-2466	3+4	2	2	2
07-620	3+4	1	2	2
08-09120	3+4	1	2	2
08-09824	3+4	1	3	2
09-05202	3+4	1	2	1
09-06540	3+4	1	1	1
09-09968	3+4	1	2	1
09-11322	3+4	1	3	1
05-10337	4+3	1	2	1
04-9936	4+3	1	2	1
06-321	4+3	1	2	2
07-6881	4+3	3	2	1
09-10713	4+3	3	3	1
09-12704	4+3	1	2	1
10-00469	4+3	1	2	2
10-01016	4+3	1	1	1
10-03044	4+3	1	1	1
08-13338	4+3	2	2	1
08-05949	4+3	1	2	2

Case No.	Gleason score	PTEN score	PLZF score	FOXO3a score
08-12900	3+5	2	2	2
07-01823	4+4	1	1	2
08-10455	4+4	1	1	1
09-06701	4+4	1	1	1
06-3742	4+5	1	1	1
08-10890	4+5	2	2	2
09-10469	5+3	2	1	1
03-5000	5+3	1	2	1
09-10825	5+4	1	1	1
09-11111	5+4	1	1	1
